# All-on-chip reconfigurable generation of scalar and vectorial orbital angular momentum beams

**DOI:** 10.1038/s41377-025-01899-7

**Published:** 2025-06-30

**Authors:** Weike Zhao, Xiaolin Yi, Jieshan Huang, Ruoran Liu, Jianwei Wang, Yaocheng Shi, Yungui Ma, Andrew Forbes, Daoxin Dai

**Affiliations:** 1 State Key Laboratory for Extreme Photonics and Instrumentation, College of Optical Science and Engineering, Zhejiang University, Hangzhou, 310058 China; 2 State Key Laboratory for Mesoscopic Physics, School of Physics, Peking University, Beijing, 100871 China; 3 School of Physics, University of the Witwatersrand, Johannesburg, South Africa

**Keywords:** Silicon photonics, Integrated optics

## Abstract

Structured light carrying angular momentum, such as spin angular momentum (SAM) and orbital angular momentum (OAM), has been at the core of new science and applications, driving the need for compact on-chip sources. While many static on-chip solutions have been demonstrated, as well as on-chip sources of free-space modes, no architecture that is fully reconfigurable in all angular momentum states and all on-chip has so far been possible. Here we report the first all-on-chip structured light generator for the creation of both scalar and vectorial angular momentum beams, facilitated through a silicon-on-insulator (SOI) chip with a silica mode multiplexer (silica chip). We selectively stimulate six linearly-polarized (LP) modes of the silica multimode bus waveguide, precisely controlling the modal powers and phases with the SOI chip. This allows us to tailor arbitrary superpositions of the mode set thus synthesizing common cylindrical vector vortex beams as well as OAM beams of controlled spin and topological charge. Our compact structured light generator exhibits high switching speed and operates across the telecom band, paving the way for applications such as optical communication and integrated quantum technologies.

## Introduction

In recent decades, structured light has captured a great deal of research interest and found a variety of applications^1–4^, including spin angular momentum (SAM) beams^5,6^, cylindrical vector (CV) beams^7,8^, orbital angular momentum (OAM) beams^9,10^, and total angular momentum (TAM) beams^11,12^, which have special spatial distributions of intensity, phase or polarization. Among them, SAM beams are associated with circular polarization and carry SAM of S=σℏ (σ = ±1) per photon, while CV beams feature a circular symmetry polarization distribution and are undetermined at the beam center where there is a polarization singularity. CV beams have shown great potential in various applications, such as plasmonic nanofocusing^13,14^, particle manipulation^15^, and high-resolution optical microscopy^16^, due to their unique focusing property. In contrast, OAM beams have a helical phase front characterized by Hilbert factor exp(*ilθ*), where *l* is the topological charge value and *θ* is the azimuthal angle^17^, resulting in a phase singularity at the beam center and an OAM of *l*ℏ per photon. The unique phase/intensity distribution of OAM beams makes them very useful for widespread applications, including mode-division multiplexing^18,19^, optical micro-manipulation^20–22^, and optical measurement^23^ to name but a few.

The myriad of applications has fueled the generation of structured light, with the bulk optick toolkit now comprising spatial light modulators^24,25^, fiber-based devices^7–9,26–30^, spiral phase plates^31^ and directly from lasers^32,33^. On-chip structured light generators come with the advantages of compactness, robustness, and versatility, and have attracted significant attention of late. A variety of silicon-on-insulator (SOI) photonic structures have been demonstrated for generating structured light^34–43^. On-chip metamaterials or metasurfaces imply a helical wavefront on the launched light by using a set of meta-atoms that offer a phase modulation of 0-2π with the principle of electric dipole resonance and generalized laws of reflection/refraction, and have been widely used to emit the OAM beams. By further utilizing the spin-orbit conversion, metamaterial also performs controllable transformation between SAM and OAM beams^39,40^. Alternatively, holographic gratings, which are compact and efficient, can couple an in-plane guided-mode to a free-space OAM mode by introducing subwavelength surface structures^41,42^. In addition, dielectric or plasmonic cavities with strong mode coupling have also been developed for on-chip OAM beam generation by using angular gratings to achieve free-space OAM beams with well-controlled topological charges from in degenerate whispering gallery modes (WGM). The different order cavity-modes at discrete resonance wavelengths map to the OAM beams carrying different topological charge values *l*^43^, and the emitting OAM order can be tuned by using an electrical heater^38^ or utilizing the strong mutual interaction of the SAM and OAM^34–37^. It should be mentioned that the aforementioned schemes are all based on the optical diffraction mechanism, and their conversion efficiency is limited to <~25% due to the downward emission to the substrate^42^. A potential solution to improve emission efficiency is coating a reflection layer on the substrate^44^ or adopting unidirectional radiation grating^45^. Furthermore, the large divergence angle of diffraction structures also makes them difficult to be coupled to OAM fibers, which thus hinders their further applications.

More recently, photonic integrated circuits (PICs) have been used to create scalar structured light modes^46,47^, a nascent direction that holds exciting future promise for structured light generators. In recent advances, the light was injected into the device, amplitude and phase modulated on-chip through a PIC, and then tailored in free-space by interference^47^ or by using a fixed metasurface^46^, producing scalar free-space modes. To realize a compact and reconfigurable approach that extends to arbitrary angular momentum beams that are controlled all on-chip (without free-space coupling) would require engineering spatial modes on the basis of on-chip waveguides or fibers and non-separably combining them with polarization, in principle allowing all-on-chip reconfigurable angular momentum creation from scalar to vectorial states of SAM and OAM light. While this is highly desirable for fully integrated functionality, it has yet to be realized^48^.

In this paper, we advance the nascent field of PICs as beam creators by demonstrating an all-on-chip reconfigurable structured light generator that produces on-demand angular momentum beams, from scalar vortex beams to vector vortex beams, all as natural fiber modes. We achieve this by using the natural modes of the silica waveguide as our basis set, controlled by six independent outputs of an SOI chip from three ports (each with two orthogonal polarizations), each reconfigurable in modal power and phase. We demonstrate this for a controlled spin and topological charge of OAM modes of order 1, as well as their vectorial combinations, including the well-known cylindrical vector vortex beams, e.g., radially and azimuthally polarised light. Our PIC architecture merges an SOI chip and a silica mode multiplexer (silica chip) for a compact device, while the all-on-chip configuration is ensured by the fiber coupled input and the subsequent on-chip excitation of scalar and vectorial OAM inside the silica multimode bus waveguide (MBW), circumventing the deleterious free-space to chip input/output coupling. Our convenient all-on-chip generation of structured light enjoys fast switching speed, a broad working bandwidth, high conversion efficiency, easy fiber-coupling (input and output), and can be extended in the future to higher OAM values and arbitrary total angular momentum by simply scaling the SOI chip output ports and MBW size without any fundamental changes to the concept or geometry.

## Results

### Concept and Implementation

**Fig. 1:**
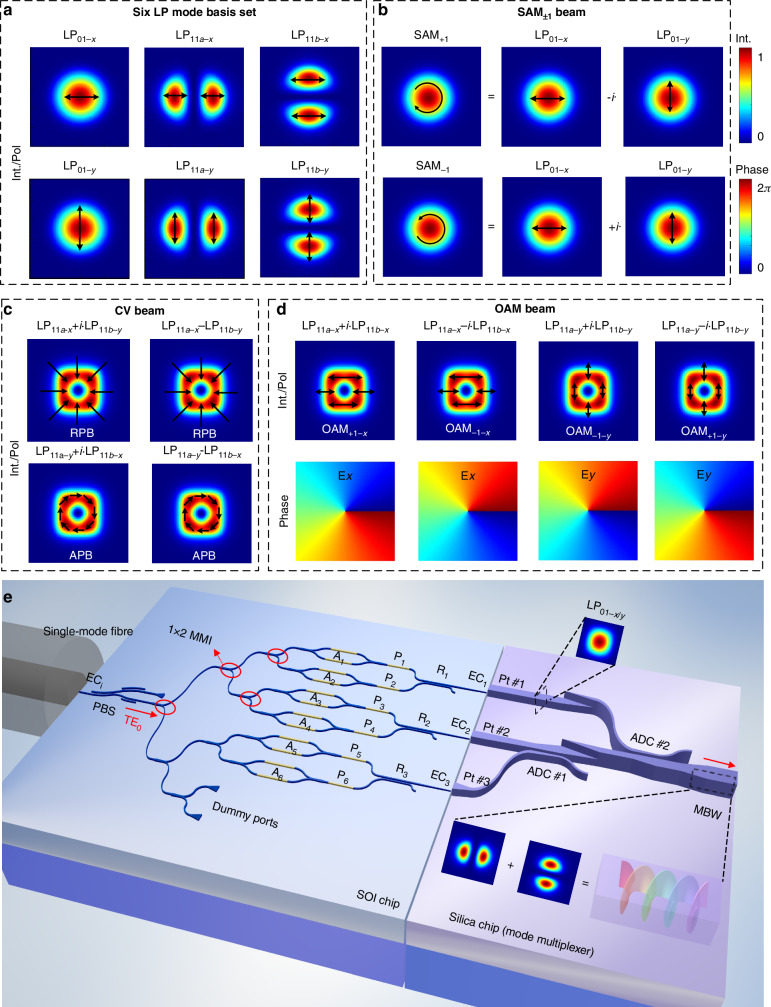
All-on-chip structured light generator. **a** Six LP mode basis sets at a few-mode fiber or a square silica waveguide, i.e., LP_01-x_, LP_01-y_, LP_11a-x_, LP_11a-y_, LP_11b-x_, and LP_11b-y_ modes. **b** Top panel: the SAM_+1_ beam (right-handed circular polarization) is synthesized by the LP_01-x_ and LP_01-y_ modes with a phase difference of π/2; bottom panel: the SAM_-1_ beam (left-handed circular polarization) is synthesized by the LP_01-x_ and LP_01-y_ modes with a phase difference of -π/2. **c** The intensity/polarization patterns of the CV beams containing RPB and APB, which both have donut intensity patterns, but are distinguished with radial and azimuthal polarization, respectively. **d** The intensity/polarization patterns and phase structures of the OAM_±1-x_ and OAM_±1-y_ beams. **e** Schematic configuration of the proposed all-on-chip structured light generator incorporating an SOI chip and a silica chip. Upper inset: the LP_01-x/y_ mode in a silica single mode waveguide; Bottom inset: the OAM_±1_ beam is synthesized as an example by using the LP_11a_ and LP_11b_ modes in a silica multimode bus waveguide (MBW). Operating principle: the light emitted by the fiber to the SOI chip is polarized with a polarization beam splitter (PBS) and then split into six TE_0_ modes with a series of 1×2 multimode-interference (MMI) 3-dB couplers. These six TE_0_ modes respectively pass through a variable optical attenuator (VOA) and a phase shifter (PS), and then are combined into three pairs of TE_0_/TM_0_ modes by three polarization splitter-rotators (PSR, R_1_-R_3_). These three pairs of TE_0_/TM_0_ mode are coupled to the LP_01-x_/ LP_01-y_ modes of three silica waveguide ports (Pt #1, Pt #2, and Pt #3) by three edge couplers (ECs, EC_1_-EC_3_). Finally, these three pairs of LP_01-x_/LP_01-y_ modes stimulate the six LP mode basis sets of the MBW through a silica mode multiplexer (containing two adiabatic directional couplers, ADC #1 and ADC #2). One can selectively synthesize the SAM_±1_, OAM_±1-x_/OAM_±1-y_ beams, as well as the CV beams by controlling the powers and phases of these six LP modes with the six VOAs (A_1_-A_6_) and PSs (P_1_-P_6_)

Our concept involves the exploitation of the SOI chip for light with controlled amplitude and phase at several output ports, using this to control the superposition of modes in a silica waveguide/fiber by edge coupling, and then finally dynamically adjusting the SOI chip to produce any desired OAM mode directly on a chip within the silica waveguide/fiber by appropriate superposition of the underlying modes. To unpack this by way of example, note that both few-mode fibers (FMFs) and square silica waveguides with appropriate cross-section parameters can support the well-known Linearly Polarised (LP) mode set, with six such LP modes (i.e., LP_01-*x*_, LP_01-*y*_, LP_11a-*x*_, LP_11a-*y*_, LP_11b-*x*_, and LP_11b-*y*_) shown in Fig. 1a. From these, it is possible to construct, by appropriate superpositions, both scalar and vectorial OAM modes of topological order 1, following^3^

1a $${{\rm{SAM}}}_{\pm 1}=({{\rm{LP}}}_{01-x}\mp {i\,\cdot\, {\rm{LP}}}_{01-y})/\sqrt{2}$$

1b $${\rm{RPB}}=({{\rm{LP}}}_{11a-x}-{{\rm{LP}}}_{11b-y})/\sqrt{2}\,{\rm{or}}\,({{\rm{LP}}}_{11a-x}+i\,\cdot \,{{\rm{LP}}}_{11b-y})/\sqrt{2}$$

1c $${\rm{APB}}=({{\rm{LP}}}_{11a-y}-{{\rm{LP}}}_{11b-x})/\sqrt{2}\,{\rm{or}}\,({{\rm{LP}}}_{11a-y}+i\,\cdot\, {{\rm{LP}}}_{11b-x})/\sqrt{2}$$

1d $${{\rm{OAM}}}_{\pm 1-x}=({{\rm{LP}}}_{11a-x}\pm i\,\cdot\, {{\rm{LP}}}_{11b-x})/\sqrt{2}$$

1e $${{\rm{OAM}}}_{\pm 1-y}=({{\rm{LP}}}_{11a-y}\pm {i\,\cdot\, {\rm{LP}}}_{11b-y})/\sqrt{2}$$

Here the subscript numbers are radial and azimuthal mode orders, a/b is the horizontal/vertical mode azimuth, and-x/-y is the horizontal/vertical polarization. Higher numbers of LP modes would naturally allow higher-order OAM modes to be engineered. Continuing with our example, we see that the desired modes can be synthesized by selectively combining these six LP modes with specific modal powers and phases, which we achieve with the PIC. For instance, the SAM_±1_ beam can be synthesized by combining the LP_01-x_ and LP_01-y_ modes with a phase difference of π/2 (for right-handed circular polarization) or -π/2 (for left-handed circular polarization) according to Eq. (1a), as shown in Fig. 1b. Note that CV beams contain the radially polarized beam (RPB) and the azimuthally polarized beam (APB), while the RPB and APB can be synthesized with the LP_11a_ and LP_11b_ modes with different polarizations by following Eqs. (1b) and (1c), respectively. The intensity/polarization distributions of the RPB and APB are clearly shown in Fig. 1c, which both have donut intensity patterns, but are distinguished with radial and azimuthal polarization, respectively. Furthermore, as given in Eqs. (1d) and (1e), the *x*- and *y*-polarized OAM_±1_ beams (i.e., OAM_±1-*x*_ and OAM_±1-y_) can be synthesized by combining the LP_11a_ and LP_11b_ modes with the same polarization. Figure 1d shows the doughnut-shaped field distributions of the OAM_±1-x_ and OAM_±1-y_ beams as well as the spiral phase structures for their E_*x*_ and E_*y*_ components.

As a summary, the aforementioned structured light can intuitively be synthesized by exciting two specific power-, polarization- and phased-controlled LP modes. Even though LP modes are well supported in low-index-contrast optical waveguides (such as silica waveguides or fibers), the manipulation of the polarization state and the phase shifting becomes very inconvenient due to the low polarization-selectivity and low thermal-tuning efficiency. In contrast, when using silicon waveguides (which have a high index contrast), it becomes very flexible to manipulate the polarization states, the power ratios, and the phase shifts of the guided modes. As a consequence, here we propose a new PIC structured light generator by incorporating an SOI chip and a silica chip, as shown in Fig. 1e. In particular, the SOI chip is used for manipulating the polarization states, the power ratios and the phase-shiftings of the guided light, so that three pairs of TE_0_/TM_0_ modes are generated with flexibly tunable power ratios and phase shifts. The silica chip is used to receive these three pairs of TE_0_/TM_0_ modes from the SOI chip and then multiplex them to the six quasi LP modes basis sets (i.e., LP_01-*x*_, LP_01-*y*_, LP_11a-*x*_, LP_11a-*y*_, LP_11b-*x*_, LP_11b-*y*_) supported in a silica MBW. With such a configuration, the power ratios and phase shifts of these six LP modes can be controlled freely by tuning the heaters on the SOI chip, and thus the SAM_±1_, OAM_±1-*x*_, and OAM_±1-*y*_ and RPB/APB beams can selectively be synthesized.

As shown in Fig. 1e, light from a single-mode fiber is coupled to the input port of the SOI chip with the assistance of silicon edge couplers (EC_*i*_) and is polarized to be the TE_0_ mode by using a polarization beam splitter (PBS)^49^. The TE_0_ mode is then split by using a 1×8 power splitter based on 1×2 multimode-inteference (MMI) 3-dB couplers in cascade. Note that there are two dummy ports used for splitting ratio and insertion loss measurement. For these six TE_0_ modes, there are six variable optical attenuators (VOAs; A_1_, A_2_, …, A_6_) to manipulate the power ratios and six phase shifters (PSs; P_1_, P_2_, …, P_6_) to manipulate the phase shifts^50^, respectively. These six TE_0_ modes with the target power ratios and phase shifts are then recombined to be three TE_0_/TM_0_ mode-pairs by using three polarization splitter-rotators (PSRs; R_1_, R_2_, R_3_)^51,52^ and output finally from Ports (Pt) #1, #2, and #3, respectively (see Methods). The SOI chip is butt coupled to the silica chip with three ECs (EC_1_, EC_2_, EC_3_), in which way the three pairs of TE_0_/TM_0_ modes in the three silicon waveguides are coupled respectively to the LP_01-x_/LP_01-y_ modes of three silica single mode waveguides. Finally, these LP_01-x_/LP_01-y_ modes are converted and multiplexed to the six LP modes basis sets (i.e., LP_01-*x*_, LP_01-*y*_, LP_11a-*x*_, LP_11a-*y*_, LP_11b-*x*_, LP_11b-*y*_) supported in the MBW, by using a polarization-insensitive silica mode multiplexer, which consists of two adiabatic directional couplers (ADCs; ADC #1, ADC #2) in cascade. In detail, the LP_01_ mode launched from Port #1 couples to the LP_11a_ mode of the MBW via ADC #2, while the LP_01_ mode launched from Port #3 couples to the LP_11a_ mode of the MBW via ADC #1 and then is rotated to the LP_11b_ mode with a mode rotator based on a dual-layer silica waveguide. Here the LP_01_ mode launched from Port #2 transmits through these two ADCs directly (see Methods). The transmission of the whole PIC structured light generator can be depicted by the transmission matrix method (Supplementary Note 1). Note that while we have outlined this concept based on the implementation of our silica MBW supporting just six modes, it can be generalized to an arbitrary mode number for both higher OAM orders and TAM control.

### Fabrication and measurement results

**Fig. 2:**
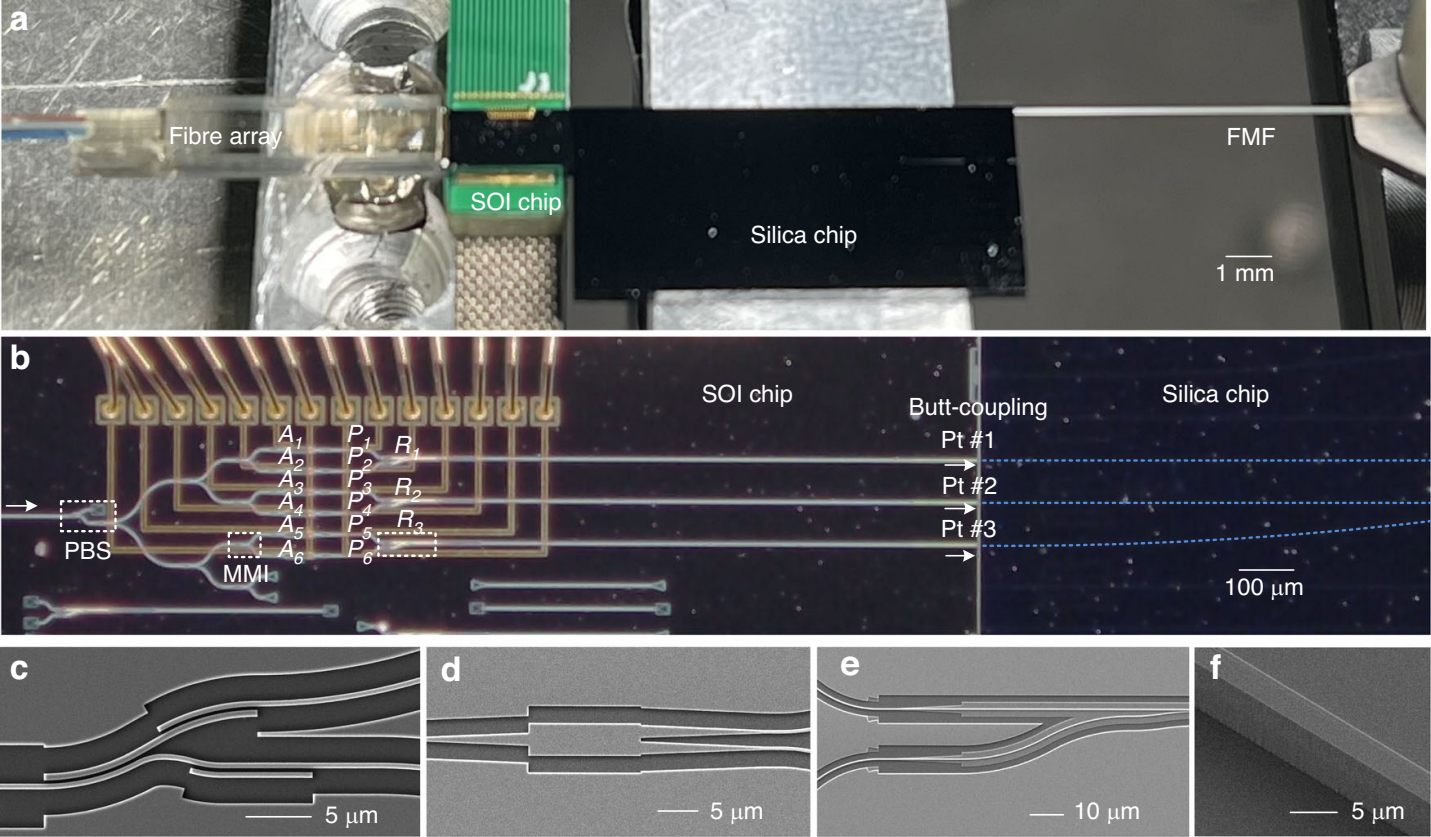
Images of the fabricated device. **a** The butt-coupled input fiber array, SOI chip, silica chip and FMF. **b** The enlarged view of the butt-coupled SOI chip and silica chip, where the PBS, MMI, VOAs (A_1_-A_6_), PSs (P_1_-P_6_), PSRs (R_1_-R_3_), three ports (Pt #1-#3) are labeled. Scanning electron microscope (SEM) images were given for the (**c**) PBS, (**d**) MMI coupler, (**e**) PSR, and (**f**) silica waveguide

The SOI and silica chips were fabricated separately with their own regular processes (Supplementary Note 2). The PBSs and PSRs fabricated on the SOI chip all have a low loss of <1 dB and crosstalk <-15 dB for both TE_0_ and TM_0_ mode channels (Supplementary Note 3). The input fiber array, SOI chip, silica chip and FMF were butt-coupled, as shown in Fig. 2a. Here an ultra-high-NA single mode fiber (HSMF) with a numerical aperture of 0.41 and a core diameter of ~2.4 μm was used for the butt coupling with the SOI chip through an EC, enabling a relatively low coupling loss of ~2 dB for the TE_0_ mode in the wavelength range of 1520–1600 nm. The coupling loss between the silicon waveguide and the 4 × 4 μm^2^ single mode silica waveguide with the help of the same EC is 0.4/1.5 dB for the TE_0_/TM_0_ modes (Supplementary Note 3). The silica mode multiplexer has a low on-chip loss of <0.8 dB and low inter-mode crosstalk of <-14.2 dB for all three modes in the broad wavelength range of 1530–1620 nm^53^. The SOI chip was wire-bonded onto a printed circuit board (PCB) for electrical control. Figure 2b shows the enlarged views of the SOI and silica chips, and the silica waveguide is labeled with a dotted line. The scanning electron microscope (SEM) images of the PBS, MMI coupler, PSR, and silica waveguide are shown in Fig. 2c–f, respectively.

### Generation of the six LP-mode basis sets

**Fig. 3:**
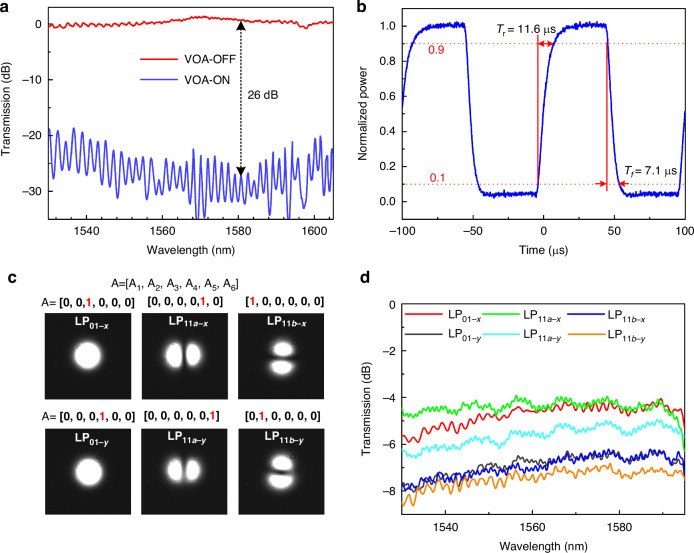
Characterization of VOAs and the excitation of the six LP mode basis sets. **a** The measured on/off transmissions of the testing VOA fabricated on the same SOI chip, showed a maximum extinction ratio of ~ 26 dB at 1580 nm. **b** The measured temporal response of the VOA, exhibits a rise time T_*r*_ of 11.6 μs and a falling time T_*f*_ of 7.1 μs. **c** Any one of six LP-modes excited individually in the MBW by controlling the VOAs to achieve the target amplitude matrix A = [A_1_, A_2_, A_3_, A_4_, A_5_, A_6_] as desired. **d** The measured transmissions from the input fiber to the output FMF when any one of these six LP modes is excited individually, show an excess loss of 4-8 dB for all six modes in a broad wavelength range of 1530-1595 nm

A multichannel voltage source (MVS) was used to power the six VOAs and six PSs, so that the power ratios and the phase shifts of these six TE_0_/TM_0_ channels can be controlled (Supplementary Note 4). Figure 3a, b gives the measurement results for the VOAs, showing an excess loss of <0.6 dB and a maximum extinction ratio of ~26 dB at the wavelength of ~1580 nm. The thermal tuning of the VOAs and PSs works with a rise time T_*r*_ of 11.6 μs and a falling time of T_*f*_ of 7.1 μs, enabling fast generation of structured light beams. This performance benchmark is comparable to state-of-the-art implementations utilizing optical antenna arrays^36^ and angular grating-integrated microring resonators^47^, which similarly employ thermo-optic modulation for dynamic OAM topological charge control. The response time of the generator can be further reduced to nanosecond lever by utilizing the carrier dispersion effect^47^ or electro-optic effect^54^. The amplitude matrix of six LP mode basis sets is given as **A** = [A_1_, A_2_, A_3_, A_4_, A_5_, A_6_], where A_m_ is the amplitude for the *m*-th mode-channel, and one has A_*m*_ = 1 or 0 by unheating or heating the *m*-th VOA (VOA_m_). As a result, appropriately controlling the VOAs enables to individually excite any one of the LP_01-x_, LP_01-y_, LP_11a-x_, LP_11a-y_, LP_11b-x_, and LP_11b-y_ modes in the MBW, as shown in Fig. 3c. A pair of silica mode multiplexers connected back to back are fabricated on the same silica chip, and the crosstalk matrix of six LP modes was obtained by measuring their port-to-port transmission. The results concluded in Table 1, show that the mode pairs have an on-chip insertion loss of less than 3.2 dB and mode crosstalk of less than 14.3 dB for all six modes at 1550 nm wavelength^53^. It can be seen that all six LP modes are generated successfully with the desired mode profiles, and the perfect azimuth orthogonality of the LP_11a_ and LP_11b_ modes verifies that the fabricated silica chip performs well. Figure 3d shows the measured transmissions from the input fiber to the output FMF when any one of these six LP modes is excited individually. It can be seen that the SOI and silica chips for the present structured light generator work well with a low excess loss of about 4–8 dB in a broad wavelength range of 1530–1595 nm. Here the excess loss contains the total on-chip losses of about 2 dB, the coupling loss of 0.4/1.5 dB between the silicon and silica chips for x-/y- polarization, and the coupling loss of ~2–4 dB between the silica chip and the few-mode fiber. It should be noted that the majority of the loss stems from the coupling losses between chips and between the chip and the fiber, with the coupling loss between the silica chip and the few-mode fiber being particularly significant. The overall efficiency, defined as the energy in each output mode at the output of the silica chip (without the coupling loss between silica chip and few-mode fiber) over the initial input energy of the source injecting the silicon chip (containing coupling loss between single mode fiber and silicon chip), is estimated to 4.9%, 3.1%, 5.5%, 2.5% and 2.6% for LP_01-x_, LP_01-y_, LP_11a-x_, LP_11a-y_, LP_11b-x_, and LP_11b-y_ modes, respectively. There is potential to further reduce the excess loss through the optimization of the chip architecture and the improvement of the fabrication process.

**Table 1:** The crosstalk matrix of the six LP modes (dB)

	LP_01-x_	LP_01-y_	LP_11a-x_	LP_11a-y_	LP_11b-x_	LP_11b-y_
LP_01-x_	−2.53	−32.73	−27.24	−35.35	−22.64	−24.21
LP_01-y_	−24.12	−1.75	−26.41	−31.22	−20.02	−27.15
LP_11a-x_	−31.24	−23.01	−2.19	−29.9	−16.45	−19.42
LP_11a-y_	−20.32	−18.46	−28.49	−1.81	−19.88	−23.02
LP_11b-x_	−22.05	−23.06	−18.05	−19.16	−3.22	−29.92
LP_11b-y_	−20.57	−23.36	−22.89	−30.97	−32.69	−2.29

### Synthesis of SAM

**Fig. 4:**
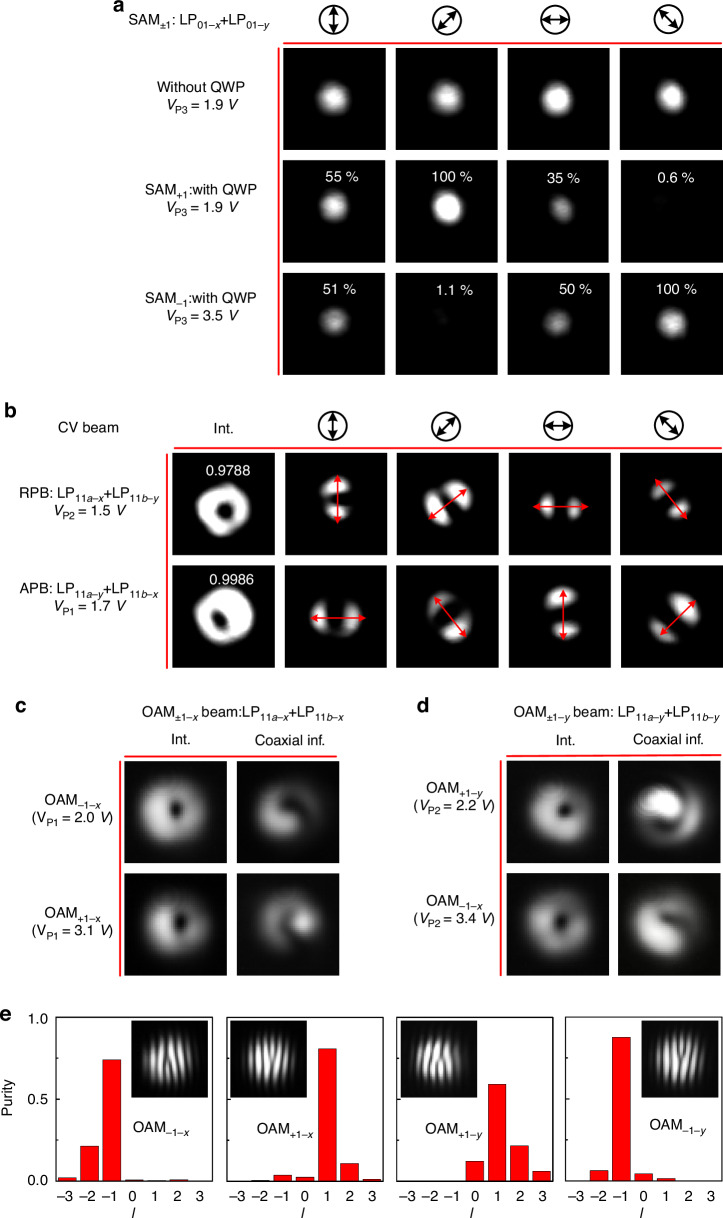
The experimental synthesis of structured light beams. **a** The SAM_±1_ beam is synthesized by incorporating the LP_01-x_ and LP_01-y_ modes and characterized with the quarter-wave plate (QWP) rotation method. Top panel: the synthesized structured light (V_P3_ = 1.9 V) passes through a polarizer with different orientations (see the black arrows), and the mode intensity remains unchanged; Middle panel: after inserting a QWP behead the polarizer, constructive interference occurs at the 45°-orientated polarizer, and destructive interference occurs at the 135°-orientated polarizer, confirming the structured light carries SAM_+1_; Bottom panel: when further increasing V_P3_ to 3.5 V, the constructive interference and the destructive interference exchange with each other, confirming the structured light carries SAM_-1_. **b** The CV beams are synthesized by incorporating the LP_11a_ and LP_11b_ modes with different polarizations. Top panel: the RPB is synthesized with the combination of LP_11a-*x*_ and LP_11b-*y*_ modes at V_P2_ = 1.5 V. It has a donut intensity pattern, and maps to an LP_11_ mode with a mode azimuth (red arrow) same as the orientation of the polarizer; Bottom panel: the APB is synthesized with the combination of LP_11a-*y*_ and LP_11b-*x*_ modes at V_P1_ = 1.7 V, and it also has a donut intensity pattern, but maps to an LP_11_ mode with a mode azimuth angle vertical to the orientation of the polarizer. **c** The OAM_±1-x_ beam is synthesized with the combination of the LP_11a-x_ and LP_11b-x_ modes. The intensity, and coaxial interference (inf.), patterns confirm the generation of OAM_-1-x_ (top panel, at V_P1_ = 2 V) and OAM_+1-x_ (bottom panel, at V_P1_ = 3.1 V). **d** The OAM_±1-y_ beam is synthesized with the combination of the LP_11a-y_ and LP_11b-y_ modes. The intensity and coaxial interference (inf.) patterns confirm the generation of OAM_-1-x_ (top panel, at V_P2_ = 2.2 V) and OAM_+1-x_ (bottom panel, at V_P2_ = 3.4 V). **e** Modal decomposition into the OAM basis for OAM_-1-x_, OAM_+1-x_, OAM_+1-y_, and OAM_-1-y_ light beam, respectively. Insets: the corresponding off-axis interference patterns. (@1550 nm)

For the generation of the SAM_±1_ beam, the matrix **A** was set to [0, 0, 1, 1, 0, 0] so that only the LP_01-*x*_ and LP_01-*y*_ modes in the MBW were excited. The modal powers of these two LP modes were balanced by tuning VOA #3 and #4, while their phase difference was tuned to be π/2 or -π/2 according to Eq. (1a) by adjusting the bias voltages V_P3_ and V_P4_ applied to the phase shifters P_3_ and P_4_. The generated SAM_±1_ beam was characterized by using a common quarter-wave plate (QWP) rotation method (Supplementary Note 4), as shown in Fig. 4a. Here the phase difference becomes π/2 and -π/2 when V_P3_ = 1.95 V and 3.5 V, respectively. When V_P3_ = 1.95 V, the mode intensity remained unchanged as the polarizer was rotated with different orientation angles of 0°, 45°, 90°, and 135° (see the black arrows) if no QWP was inserted. In contrast, when a QWP with a horizontal fast axis was placed in front of the polarizer, one observed constructive and destructive interference when the polarizer was 45°- and 135°-orientated, respectively, as shown in the middle panel of Fig. 4a, indicating that the generated beam carries SAM_+1_ as expected. When further increasing V_P3_ to 3.5 V, constructive and destructive interference respectively happens when the polarizer is 135°- and 45°-orientated, as shown in the bottom panel of Fig. 4a, indicating the SAM_-1_ beam is generated as desired. Based on the transmittance labeled in Fig. 4a, the purity of the SAM_±1_ beams is deduced to be larger than 0.989. Note that the output beam’s polarization state can traverse the Poincaré sphere by appropriately setting A_3_, A_4_ and V_P3/4_.

### Synthesis of CV beams

When matrix **A** is set to be [0, 1, 0, 0, 1, 0], the LP_11a-x_ and LP_11b-y_ modes in the MBW are excited simultaneously, and their phase difference can be tuned to 0 or -π/2, in which case the RPB beam is generated according to Eq. (1b). As shown by the top panel in Fig. 4b, the synthesized mode field has a donut-shaped intensity when V_P2_ = 1.5 V. When a polarizer with rotated orientation angles is inserted before the CCD camera, a series of LP_11_ mode fields with different azimuth are achieved (red arrow) and the LP_11_ mode’s azimuth is perfectly consistent with the polarizer orientation angles, verifying that the generated CV beam is RPB. In contrast, matrix **A** should be set as [1, 0, 0, 0, 0, 1] to synthesize the APB according to Eq. (1c), in which way the LP_11a-*y*_ and LP_11b-*x*_ modes are excited simultaneously. A donut intensity pattern appears when V_P1_ = 1.7 V, as shown in the bottom panel of Fig. 4b, while the azimuth of the LP_11_ mode is perpendicular to the polarizer’s transmission axis, as expected. The nonideal intensity pattern of the CV beam may be due to the unfavorable orientation of the modal basis, and one can optimize the design of the silica mode multiplexer to achieve a better LP mode basis set. Moreover, the unbalanced power ratio and phase difference of the two basis modals can also induce this result. To address this problem, one can optimize the architecture of the generator, thus decreasing and balancing the insertion loss of each channel on the one hand. On the one hand, more attention should be paid to the thermal management of the silicon chip, thus controlling the power and phase of these mode channels accurately. Nevertheless, the vector quality factor can be extracted using Concurrence as a measure^55^, determining the non-separability of the field. With this approach, we find purity values of 0.9788 and 0.9986 for the two vectorial modes as labeled in Fig. 4b, respectively.

### Synthesis of OAM beams

To generate OAM_±1_ beams, the LP_11a_ and LP_11b_ modes with the same polarization should be stimulated according to Eqs. (1d) and (1e). To verify the phase information of the generated OAM beams, an interference measurement method was used (Supplementary Note 4). For the synthesis of OAM_+1-x_, the LP_11a-*x*_ and LP_11b-*x*_ modes should be excited simultaneously and accordingly matrix **A** is set as [1, 0, 0, 0, 1, 0]. As shown in the top panel of Fig. 4c, one observes a donut-shaped intensity pattern and a clockwise helical coaxial interference pattern when setting V_P1_ = 2.0 V for achieving the desired π/2 phase difference between the LP_11a-x_ and LP_11b-x_ modes, which indicates that the OAM_-1-x_ beam is synthesized successfully. By further increasing the applied voltage V_P1_ to 3.1 V thus adjusting the phase difference to -π/2, one can also generate the OAM_+1-x_ beam, as shown in the bottom panel of Fig. 4c. It can be seen that the generated OAM_+1-x_ beam has a donut-shaped intensity pattern and an anti-clockwise helical coaxial interference pattern. Similarly, OAM_+1-y_ and OAM_-1-y_ beams can also be synthesized with the LP_11a-*y*_ and LP_11b-*y*_ modes by making the phase difference be -π/2 and π/2 when setting V_P2_ = 2.2 and 3.4 V, respectively, as shown in Fig. 4d. To determine the modal purity we perform a modal decomposition into the OAM basis^56^, with the results shown in Fig. 4e for OAM_-1-x_, OAM_+1-x_, OAM_+1-y_, and OAM_-1-y_ light beam, respectively. The insets show the corresponding off-axis interference patterns. We see the majority of the modal power in the desired modal, with small cross-talk contributions from neighboring modes.

### Extension to TAM

**Fig. 5:**
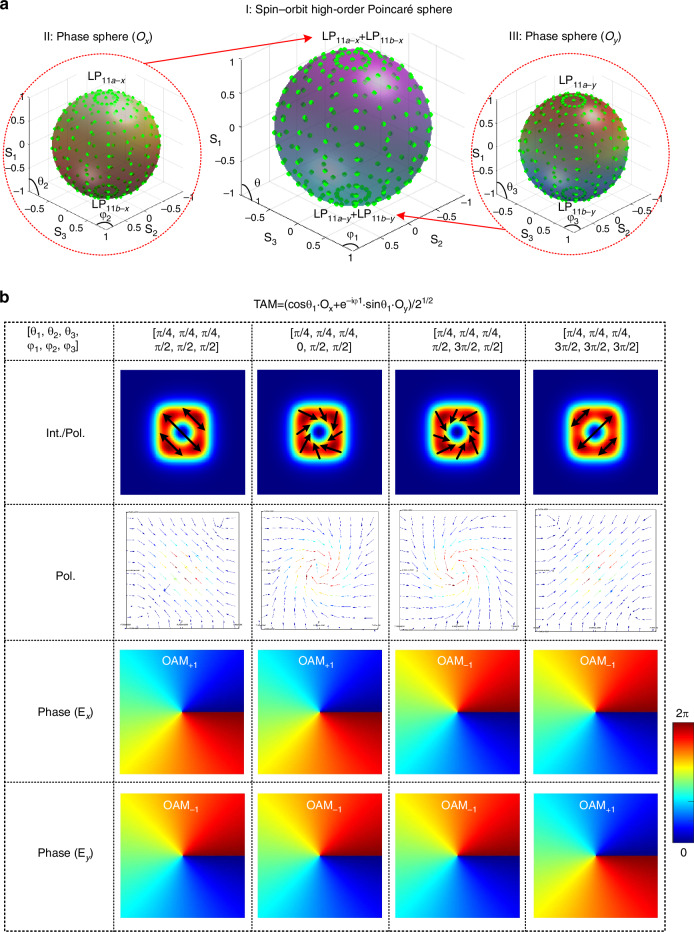
The synthesis of the TAM beams. **a** Schematic of the synthesis of the TAM beam by stimulating LP_11a-*x*_, LP_11a-*y*_, LP_11b-*x*_, LP_11b-*y*_ modes, spin-orbit high-order Poincaré multi-sphere (I) show the polarization sphere of the TAM beam, while inset (II) and (III) show the phase sphere of the O_x_ and O_y_ components of the TAM beam. **b** The intensity/polarization patterns, phase structure of the E_*x*_ component, and phase structure of the E_*y*_ component of the synthesized typical TAM beam with different phases [*θ*_1_, *θ*_2_, *θ*_3_, *φ*_1_, *φ*_2_, *φ*_3_]. When [*θ*_1_, *θ*_2_, *θ*_3_, *φ*_1_, *φ*_2_, *φ*_3_]=[π/4, π/4, π/4, π/2, π/2, π/2], the structured light has a donut intensity, 135° linear polarization and carries OAM_+1-x_ and OAM_+1-y_; When [*θ*_1_, *θ*_2_, *θ*_3_, *φ*_1_, *φ*_2_, *φ*_3_]=[π/4, π/4, π/4, 0, π/2, π/2], the structured light has a donut intensity, clockwise-vortex polarization and carries OAM_+1-x_ and OAM_+1-y_; [*θ*_1_, *θ*_2_, *θ*_3_, *φ*_1_, *φ*_2_, *φ*_3_]=[π/4, π/4, π/4, π/2, 3π/2, π/2], the structured light has a donut intensity, anticlockwise-vortex polarization, and carries OAM_-1-x_ and OAM_-1-y_; When [*θ*_1_, *θ*_2_, *θ*_3_, *φ*_1_, *φ*_2_, *φ*_3_]=[π/4, π/4, π/4, 3π/2, 3π/2, 3π/2], the structured light has a donut intensity, 45° linear polarization, and carries OAM_-1-x_ and OAM_+1-y_

Theoretically, the proposed all-on-chip structured light generator can synthesize a TAM beam that carries both OAM and SAM. A TAM beam is usually described with a spin-orbit higher-order Poincaré (HOP) multi-sphere as shown in Fig. 5a^37^, in which the basis states are more general orthogonal states that incorporate both SAM and OAM^12,57^, and the S parameters (S_1_, S_2_, S_3_) are defined as the Stokes parameter of Poincaré sphere^50^. In our case, a TAM beam can be described as follows

2 $${\rm{TAM}}=(\cos {\uptheta }_{1}\cdot {{\rm{O}}}_{x}+{e}^{-i{\upphi }_{1}}\cdot \,\sin {\uptheta }_{1}\cdot {{\rm{O}}}_{{\rm{y}}})/\sqrt{2},\,({\uptheta }_{1}\in [0,0.5\pi ],{\upphi }_{1}\in [0,2\pi ])$$

in which

2-a $${{\rm{O}}}_{x}=(\cos {{{\uptheta }}}_{2}\cdot {{\rm{LP}}}_{11a-x}+{e}^{-i{\upphi }_{2}}\cdot \,\sin {{{\uptheta }}}_{2}\cdot {{\rm{LP}}}_{11b-x})/\sqrt{2},\,({{{\uptheta }}}_{2}\in [0,0.5{{\uppi }}],{\upphi }_{2}\in [0,2{{\uppi }}])$$

2-b $${{\rm{O}}}_{y}=(\cos {{{\uptheta }}}_{3}\cdot {{\rm{LP}}}_{11a-y}+{e}^{-i{\upphi }_{3}}\cdot \,\sin {{{\uptheta }}}_{3}\cdot {{\rm{LP}}}_{11b-y})/\sqrt{2},\,({{{\uptheta }}}_{3}\in [0,0.5{{\uppi }}],{\upphi }_{3}\in [0,2{{\uppi }}])$$

Therefore, a TAM beam can be synthesized with four LP_11_ modes (i.e., LP_11a-*x*_, LP_11a-*y*_, LP_11b-*x*_, LP_11b-*y*_) and carries six parameters (i.e., *θ*_1_, *φ*_1_*, θ*_2_, *φ*_2_*, θ*_3_, and *φ*_3_). More details about these parameters are explained below.

1. *θ*_2_ and *φ*_2_ decide the power ratio and phase difference between the LP_11a-*x*_ and LP_11b-*x*_ modes, determining the phase structure of the E_*x*_ component of the TAM beam, as shown in Fig. 5a sphere II.
2. *θ*_3_ and *φ*_3_ decide the power ratio and phase difference between the LP_11a-*x*_ and LP_11b-*x*_ modes, determining the phase structure of the E_*y*_ component of the TAM beam, as shown in Fig. 5a sphere III.
3. *θ*_1_ and *φ*_1_ decide the power ratio and phase difference between the O_*x*_ and O_*y*_ components, determining the polarization state of the TAM beam, as shown in Fig. 5a sphere I.

As an example, Fig. 5b shows some of the synthesized TAMs, showing their intensity/polarization patterns, and the phase structures of their E_x_ and E_y_ components. In these examples, the *θ*_1_ = *θ*_2_ = *θ*_3_ = π/4, thus all four LP_11_ modes are excited equally. And the phase terms *φ*_2_ and *φ*_3_ are chosen to be π/2 or 3π/2 so that both E_*x*_ and E_*y*_ components carry OAM_±1_; while *φ*_1_ is appropriately chosen to produce the linear polarization (e.g., [*θ*_1_, *θ*_2_, *θ*_3_, *φ*_1_, *φ*_2_, *φ*_3_]=[π/4, π/4, π/4, π/2, π/2, π/2] or =[π/4, π/4, π/4, 3π/2, 3π/2, 3π/2]) or the vortex-like polarization (e.g., [*θ*_1_, *θ*_2_, *θ*_3_, *φ*_1_, *φ*_2_, *φ*_3_]=[π/4, π/4, π/4, 0, π/2, π/2] or =[π/4, π/4, π/4, π/2, 3π/2, π/2]), respectively. For the structured light with [*θ*_1_, *θ*_2_, *θ*_3_, *φ*_1_, *φ*_2_, *φ*_3_]=[π/4, π/4, π/4, 0, π/2, π/2] considered as an example, it has clockwise vortex-like polarization distribution, and the components of E_x_ and E_y_ carry OAM_+1_, OAM_-1_, respectively. By appropriately choosing the phase differences [*θ*_1_, *θ*_2_, *θ*_3_, *φ*_1_, *φ*_2_, *φ*_3_], one can traverse the entire HOP multi-sphere and synthesize the desired light beams carrying OAM_±1-x_, OAM_±1-y_, and SAM^41^, simultaneously. To generate the aforementioned TAM beams, the powers and phases of these four LP_11_ modes should be accuracy adjusted. However, it is difficult to achieve due to the thermal crosstalk. One can adopt the structures of air trenches, free-standing^58^ to increase the thermal efficiency and thus decrease mode crosstalk.

## Discussion

In summary, we have demonstrated an all-on-chip reconfigurable structured light generator by incorporating an SOI chip with a silica chip. Our work advances the nascent field of PICs for structured light creation, showing how to both create and control the light all on-chip without the clumsy external free-space conversion (which negates the very benefit of starting on-chip in the first place). We demonstrate its power by going beyond the state-of-the-art and showing full angular momentum control, from scalar orbital angular momentum (OAM) to vectorial combinations, made possible by full polarization control. Not only do we create this on-chip, but we also keep it there, with direct control and delivery through a waveguide. Further, our device has a fiber input and fiber output for a truly integrated, compact and reconfigurable solution, showing excellent performance in modal spectrum, wavelength spectrum and speed. We believe that this makes our solution idea for applications such as mode division multiplexing in fiber, fiber sensing with structured light and on-chip quantum technologies based on the spatial modes of light.

The proposed structured light generator can synthesize more complex total angular momentum beams by stimulating four LP_11_ modes simultaneously. While to scale to a higher order OAM state, one should design a silica mode multiplexer supporting higher order LP modes such as LP_21a_, LP_21b_, and LP_20_ modes, by using the cascaded vertical coupler^59^ or mode rotator based on a trenched waveguide^60^. Moreover, the thermal crosstalk increases as the number of activated phase shifters, which results in the inaccurate power and phase difference of these model basis. To address this issue, structures like air trenches and free-standing^58^ can be adopted. These structures can enhance the thermal efficiency of the phase shifter, thereby reducing mode crosstalk.

Compared with the methods reported previously on-chip generating structured light, the scheme proposed here shows a prominent advantage of versatility, broad bandwidth, and high conversion efficiency. Since the silica MBW can be butt-coupled efficiently to an FMF with a low coupling loss of <2 dB or less for all six LP modes by using the proposed multimode segmented waveguide^61^, the structured light beams synthesized by the present all-on-chip generator can be guided into an OAM fiber conveniently for remote applications. Regarding mode fidelity characterization, while current experimental limitations prevent comprehensive modal purity analysis across the entire structured light spectrum, we have established a theoretical framework correlating output fidelity with two fundamental parameters: intrinsic mode purity of the LP basis states, and precision in amplitude and phase control of modal superposition. Our experimental measurements confirm intermodal crosstalk suppression below -14.2 dB for all six LP modes^53^. Through rigorous optimization of relative power ratios and phase differences among constituent modes, as demonstrated in our previous work^50^, our scheme expects to achieve a high theoretical mode fidelity. For further development, efforts should be concentrated on optimizing and simplifying the chip architecture. This can be achieved by employing the structures of a variable power splitter^62^, 2-D grating coupler^63^ and high-efficiency phase shifter^58^, thus decreasing the insertion loss and thermal crosstalk. Through these means, the insertion loss and thermal crosstalk can be significantly reduced. Once the chip architecture is enhanced, it becomes possible to delve into the generation of more intricate TAM beams and skyrmions, as indicated in reference^64,65^. Additionally, these various structured light beams can be utilized in communication^63,66^ and particle manipulation^67^.

## Methods

### Silicon photonic chip design

**Fig. 6:**
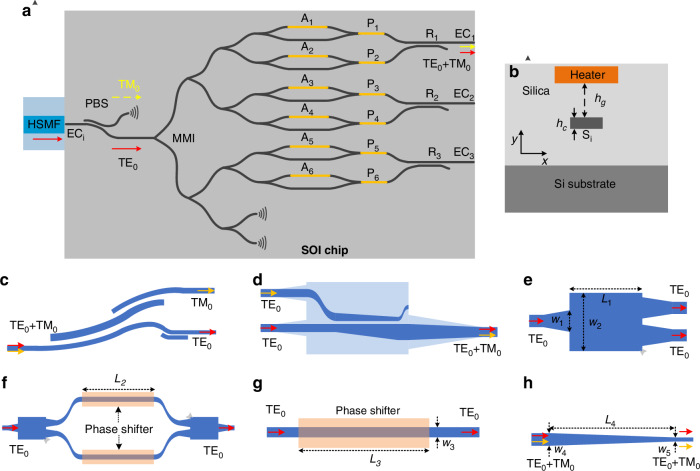
Silicon photonic chip. **a** Schematic configuration of the SOI chip, which consists of a polarization beam splitter (PBS), seven 1×2 3-dB multimode interference (MMI) couplers, six variable optical attenuators (VOAs; A_1_, A_2_, …, A_6_), six phase shifters (PSs; P_1_, P_2_, …, P_6_), three polarization-splitter rotators (PSRs; R_1_, R_2_, R_3_), and four edge couplers (EC; EC_1_, EC_2_, EC_3_). **b** The silicon waveguide cross-section. The schematic configuration of the (**c**) PBS, (**d**) PSR, (**e**) 1×2 MMI coupler, (**f**) VOA, (**g**) PS, and (**h**) EC

Figure 6a shows the schematic configuration of the SOI chip that produces three pairs of TE_0_/TM_0_ modes whose power ratios and phase shifts can be tuned thermally. The SOI chip consists of a PBS, seven 1 × 2 MMI 3-dB couplers, six variable optical attenuators (VOAs; A_1_, A_2_, …, A_6_), six phase shifters (PSs; P_1_, P_2_, …, P_6_), three PSRs (R_1_, R_2_, R_3_), and four ECs (EC_*i*_, EC_1_, EC_2_, EC_3_). Among them, 1 × 2 MMIs, VOAs, phase shifters, and ECs are relatively robust and have a fabrication tolerance of tens of nanometers. However, the PBS and PSR used for polarization manipulation have more critical fabrication requirements. To address this, we have adopted new structures (such as bent directional coupler and adiabatic coupler)^49^ and mechanisms (such as mode hybridity)^51^ to increase the bandwidth, fabrication tolerance, and polarization extinction ratio of the PBS and PSR. Therefore, the designed silicon chip has good performance and fabrication reproducibility. Figure 6b shows the cross-section of the silicon photonic waveguide, which has a 220-nm-thick silicon core layer surrounded by silica, and a metal micro-heater is located on the top with a separation of *h*_*g*_ = 1.5 μm to balance the heating efficiency and optical absorption loss. The PBS used here is designed based on the bent directional coupler as shown in Fig. 6c proposed in^49^. The PSR used here works on the principle of mode hybridness of the tapered ridge waveguide, and a directional coupler is further used to separate the TE_0_ and TM_0_ modes, as shown in Fig. 6d^51,52^. The 1 × 2 3-dB coupler is designed based on MMI as shown in Fig. 6e. The VOAs are designed based on a symmetric 1 × 1 Mach-Zender interferometer (MZI) as shown in Fig. 6f. The designed phase shifters are shown in Fig. 6g, which utilizes the effective thermo-optical coefficient difference between the TE_0_ and TM_0_ modes in a silicon photonic waveguide, and the waveguide width of 0.45 μm is chosen to balance the thermal efficiency and transmission loss^50^. The EC based on an inverse taper structure at the two ends of the SOI is used for the butt coupling between the SOI chip and HSMF or silica single mode waveguide, as shown in Fig. 6h. The inverse taper is linearly tapered from 0.45 to 0.16 μm, thus well matching the mode field of the HSMF or silica single mode waveguide. The key parameters of the designed silicon components are summarized in Table 2.

**Table 2:** The key parameters of the designed silicon photonic devices

Parameters	*w*_1_	*w*_2_	*w*_3_	*w*_4_	*w*_5_	*L*_1_	*L*_2_	*L*_3_	*L*_4_
Value (μm)	1.6	4	0.45	0.45	0.16	13.75	50	50	180

### Silica mode multiplexer design

**Fig. 7:**
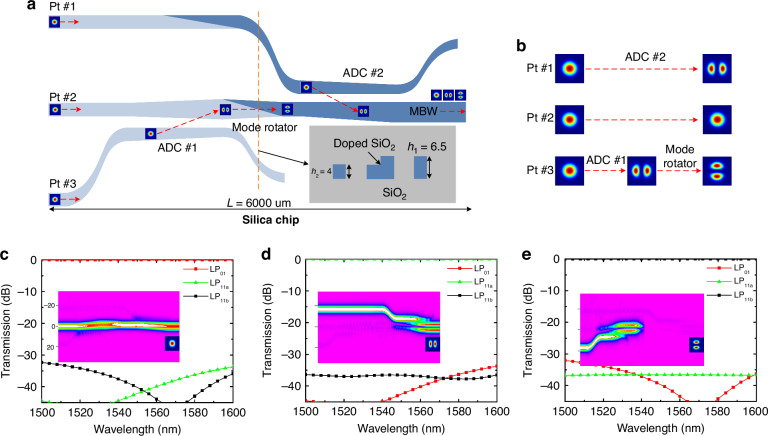
Silica chip. **a** Schematic configuration of the silica mode multiplexer, consisting of three input ports (Pt #1, Pt #2, Pt #3), two adiabatic directional couplers (ADC #1 and ADC #2), a mode rotator, and an output silica multimode bus waveguide (MBW). The inset shows the cross-section of the silica waveguide, it has a doped silica core and a pure silica cladding, while the silica single mode waveguide and multimode waveguide are designed with the core heights of *h*_2_ = 4 μm and *h*_1_ = 6.5 μm, respectively. **b** Operation principle: the LP_01_ mode launched into Pt #1 couples to the LP_11a_ mode of the MBW via ADC #2; the LP_01_ mode launched into Pt #2 passes through the two ADCs directly; the LP_01_ mode launched into Pt #3 is coupled to the LP_11a_ mode of the MBW via ADC #1 first and is then rotated to the LP_11b_ mode with a mode rotator based on the tilt-etched dual-layer waveguide. Simulated transmissions when light launched into **(c)** Pt #2, **(d)** Pt #1, and **(e)** Pt #3, the insets show the corresponding light propagation at 1550 nm

The three-channel silica mode multiplexer consists of two cascaded adiabatic directional couplers (ADC #1, ADC #2), a mode rotator and has a total length L = 6000 μm as shown in Fig. 7a^53^. The silica mode multiplexer is polarization-insensitive due to the low birefringence and low index contrast. The inset of Fig. 7a shows the cross-section of the silica waveguide, whose index contrast is about 1.5%. In particular, the silica waveguides are designed with two different heights, i.e., *h*_1_ = 6.5 μm and *h*_2_ = 4, to realize efficient conversion of the LP_01_ and LP_11b_ modes which have different mode-field symmetry^68^. The core sizes of the single mode input waveguide and multimode output waveguide are 4.0 × 4.0 μm^2^ and 6.5 × 6.5 μm^2^, respectively. At the output port, the silica multimode bus waveguide (MBW) can couple to the FMF with high efficiency and low crosstalk for the applications. The operation principle of the mode multiplexer is shown with details in Fig. 7b. Here the LP_01_ mode launched into Pt #1 couples to the LP_11a_ mode of the MBW via ADC #2; the LP_01_ mode launched into Pt #2 passes through the two ADCs directly; meanwhile, the LP_01_ mode launched into Pt #3 couples to the LP_11a_ mode of the MBW via ADC #1 first and then rotates to LP_11b_ mode with a mode rotator based on the tilt-etched dual-layer waveguide. The simulated transmissions for the lights launched into Pt #2, Pt #3, and Pt #1 are shown in Fig. 7c–e, respectively, while the insets show the corresponding light propagation for the operation at the wavelength of 1550 nm. The simulation results show the designed mode multiplex has an ultra-low loss less than 0.04 dB and crosstalk less than -32 dB for the LP_01_, LP_11a_, and LP_11b_ modes in the wavelength range of 1500-1600 nm. The ultra-low loss and low crosstalk can be attributed to the principle of adiabatic mode evolution in the designed ADCs. The currently proposed structured light beam generator integrates a silicon chip with a silica chip, it is not particularly compact. Future work can consider replacing the silica mode multiplexer with a two-dimensional grating coupler^63^ or grating antenna array^66^, thus integrating all the modules on one chip.

## Supplementary information

Supplementary information for All-on-chip reconfigurable generation of scalar and vectorial orbital angular momentum beams

## Data Availability

All data are available in the main text or the supplementary materials.
