# Supplementary material for: All-on-chip reconfigurable generation of scalar and vectorial orbital angular momentum beams
Source: Light Sci Appl. 2025 Jun 30;14:227. doi: 10.1038/s41377-025-01899-7 (PMC12209453; doi:10.1038/s41377-025-01899-7)
Supplement: Supplementary file 1 — Supplementary information for All-on-chip reconfigurable generation of scalar and vectorial orbital angular momentum beams [file 41377_2025_1899_MOESM1_ESM.docx]

## Supplementary information for All-on-chip reconfigurable generation of scalar and vectorial orbital angular momentum beams

Weike Zhao^1,#^, Xiaolin Yi^1,#^, Jieshan Huang^2^, Ruoran Liu^1^, Jianwei Wang^2^, Yaocheng Shi^1^, Yungui Ma^1^, Andrew Forbes^1,3*^ and Daoxin Dai^1,*^

*^1^State Key Laboratory for Extreme Photonics and Instrumentation, College of Optical Science and Engineering, Zhejiang University, Hangzhou 310058, China.*

*^2^State Key Laboratory for Mesoscopic Physics, School of Physics, Peking University, Beijing 100871, China.*

*^3^School of Physics, University of the Witwatersrand, Johannesburg, South Africa.*

*****Corresponding author: [dxdai@zju.edu.cn](mailto:dxdai@zju.edu.cn), [Andrew.Forbes@wits.ac.za](mailto:Andrew.Forbes@wits.ac.za)

^#^These authors contributed equally to this work: Weike Zhao, Xiaolin Yi

This PDF file includes: Supplementary Notes (Notes 1-4)

SUPPLEMENTARY INFORMATION FIGURES (Figs. S1-S3)

#### SUPPLEMENTARY NOTE 1: Transmission matrix

The transmission of the structured light generator can be depicted by the following transmission matrix:

$E_{out}=C_{P}C_{A}E_{in}$ (1)

in which E_in_ is the excited six LP modes basis sets, C_A_ is the amplitude matrix controlled by the six VOAs, and C_p_ is the phase matrix controlled by the six PSs. They are given as:

$E_{in}={[\begin{matrix} \mathrm{LP}_{11a-x} & \mathrm{LP}_{11a-y} & \mathrm{LP}_{01-x} & \mathrm{LP}_{01-y} & \mathrm{LP}_{11b-x} & \mathrm{LP}_{11b-y} \end{matrix}]}^{T}$ (1-a)

$C_{A}=\left[ \begin{matrix} A_{1} & 0 & 0 & 0 & 0 & 0 \\ 0 & A_{2} & 0 & 0 & 0 & 0 \\ 0 & 0 & A_{3} & 0 & 0 & 0 \\ 0 & 0 & 0 & A_{4} & 0 & 0 \\ 0 & 0 & 0 & 0 & A_{5} & 0 \\ 0 & 0 & 0 & 0 & 0 & A_{6} \end{matrix} \right]$ (1-b)

$C_{p}=\left[ \begin{matrix} e^{-i\varphi_{1}} & e^{-i\varphi_{2}} & e^{-i\varphi_{3}} & e^{-i\varphi_{4}} & e^{-i\varphi_{5}} & e^{-i\varphi_{6}} \end{matrix} \right]$ (1-c)

#### SUPPLEMENTARY NOTE 2: Fabrication

The SOI wafer used here has a 3-μm-thick buffer layer and a 220-nm-thick top-silicon core layer. The processes of electron beam lithography (EBL) and inductively coupled plasma (ICP) were used to form the bi-level ridge waveguides with a slab thickness of 70 nm. A 2.3-μm-thick silica upper cladding was deposited with the plasma-enhanced chemical vapor deposition (PECVD) process, and the 300-nm Cr/Ti metal layer was embedded into the cladding as the heater.

The silica chip was fabricated with a silica wafer with a silicon substrate, a 10-μm-thick silica buffer layer, and a 6.5-μm-thick doped silica-core layer. Here the index contrast of silica waveguides is about 1.5%. The silica core layer was etched with the Cr mask by using the inductively coupled plasma (ICP) process. Finally, a 15-μm silica upper cladding was formed by using the flame hydrolysis deposition (FHD) technology.

#### SUPPLEMENTARY NOTE 3: Butt-coupling loss and PBS, PSR performances

The HSMF and SOI waveguide coupling was achieved with a silicon EC based on an inverse taper waveguide. The measured coupling loss for each HSMF-SOI facet is shown in Fig. S1(a), and it is 2 dB for the TE_0_ mode in the 1520-1600 nm wavelength range. The coupling between the SOI waveguide and a 4×4 μm^2^ single mode silica waveguide is also achieved with the same EC. Fig. S1(b) shows the measured SOI-Silica butt-coupling loss, which is about 0.4/1.5 dB for the TE_0_/TM_0_ modes in the wavelength range around 1560 nm.


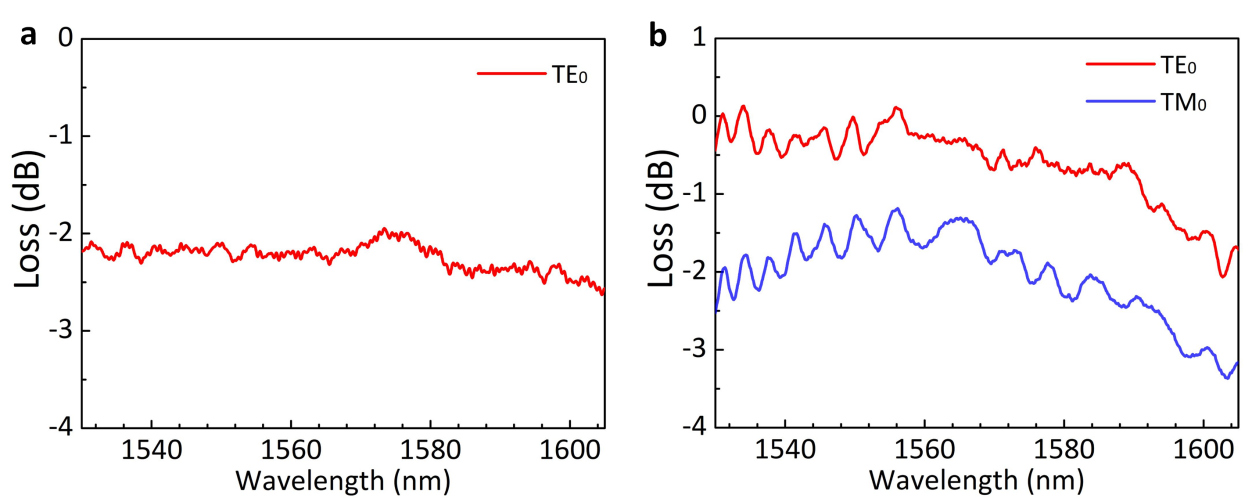


**Figure S1.** **(a)** The measured butt-coupling loss for the TE_0_ mode between an HSMF and a silicon waveguide; **(b)** The measured butt-coupling losses for TE_0_/TM_0_ modes between a silicon waveguide and a silica waveguide.

The measure results for the testing silicon PBS and PSR fabricated on the same chip are shown in Fig. S2(a, b), respectively. The PBS has a low loss of <1 dB and low crosstalk <-15 dB for both the TE_0_ and TM_0_ modes. The PSR has a low loss of <1 dB and low crosstalk <-15 dB for both the TE_0_ and TM_0_ modes.

**
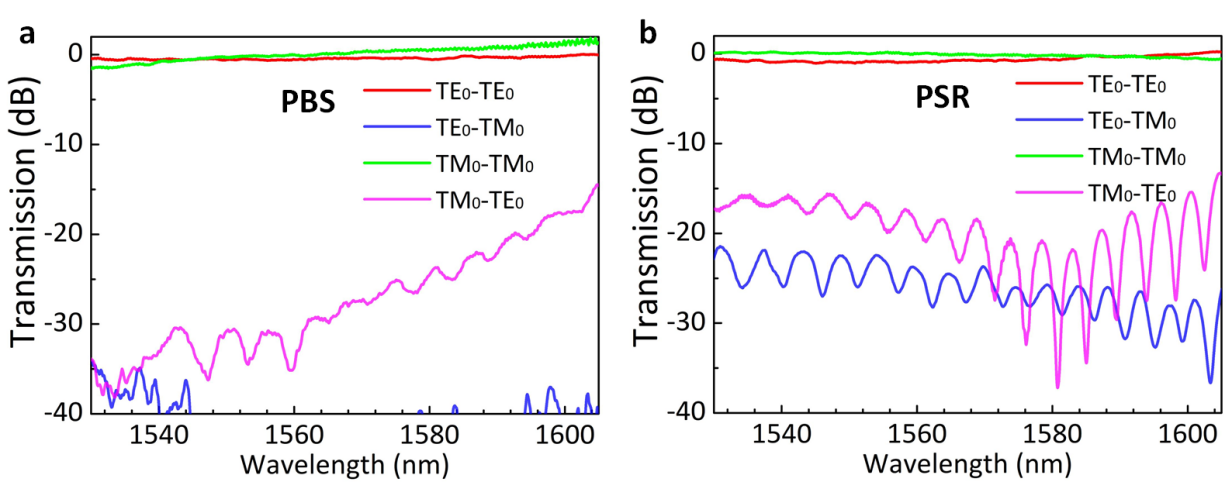
**

**Figure S2.** The measured transmissions for **(a)** the PBS, **(b)** the PSR.

#### SUPPLEMENTARY NOTE 4: Measurement

Figure S3(a) shows the experimental setup for mode field detection. Here a tunable laser (TL) @1550 nm and a fibre polarization controller (PC) connected with an HSMF were used at the input side to be butt-coupled efficiently to the SOI chip, while the SOI chip was then butt-coupled to the silica chip. Finally, the light output from the silica MBW was collimated by a 20× objective, then passed through a QWP (with a horizontal fast axis), a polarizer, and was finally captured with a CCD camera. A multichannel voltage source (MVS) was used to power the six VOAs and six PSs, so that the power ratios and the phase shifts of these six TE_0_/TM_0_ channels can be controlled. Fig. S3(b) shows the experimental setup for measuring the transmission of generated six LP-mode basis sets. Here amplified spontaneous emission (ASE) was used as a light source, and the output LP modes were received with a few-mode fibre (FMF) and sent into an optical spectrum analyzer (OSA).

The interference setup for detecting the synthesized OAM light beam is shown in Fig. S3(c). Here light from a tunable laser was divided into two parts with a power ratio of 50%:50% through a fibre 3-dB coupler. One part was sent into the SOI chip and used as the signal beam, and the other one was used as the reference Gaussian beam for interference. The signal light passed through the chips and finally was expanded with a 20× Objective, while the reference Gaussian beam was collimated with a fibre collimator. When the signal light and the reference Gaussian beam were combined with a nonpolarizing beam splitter (NPBS), and the generated interference pattern was then captured with a CCD camera. The power ratio of these two routes was balanced with two optical attenuators, and their polarization states were calibrated with two fibre PCs.


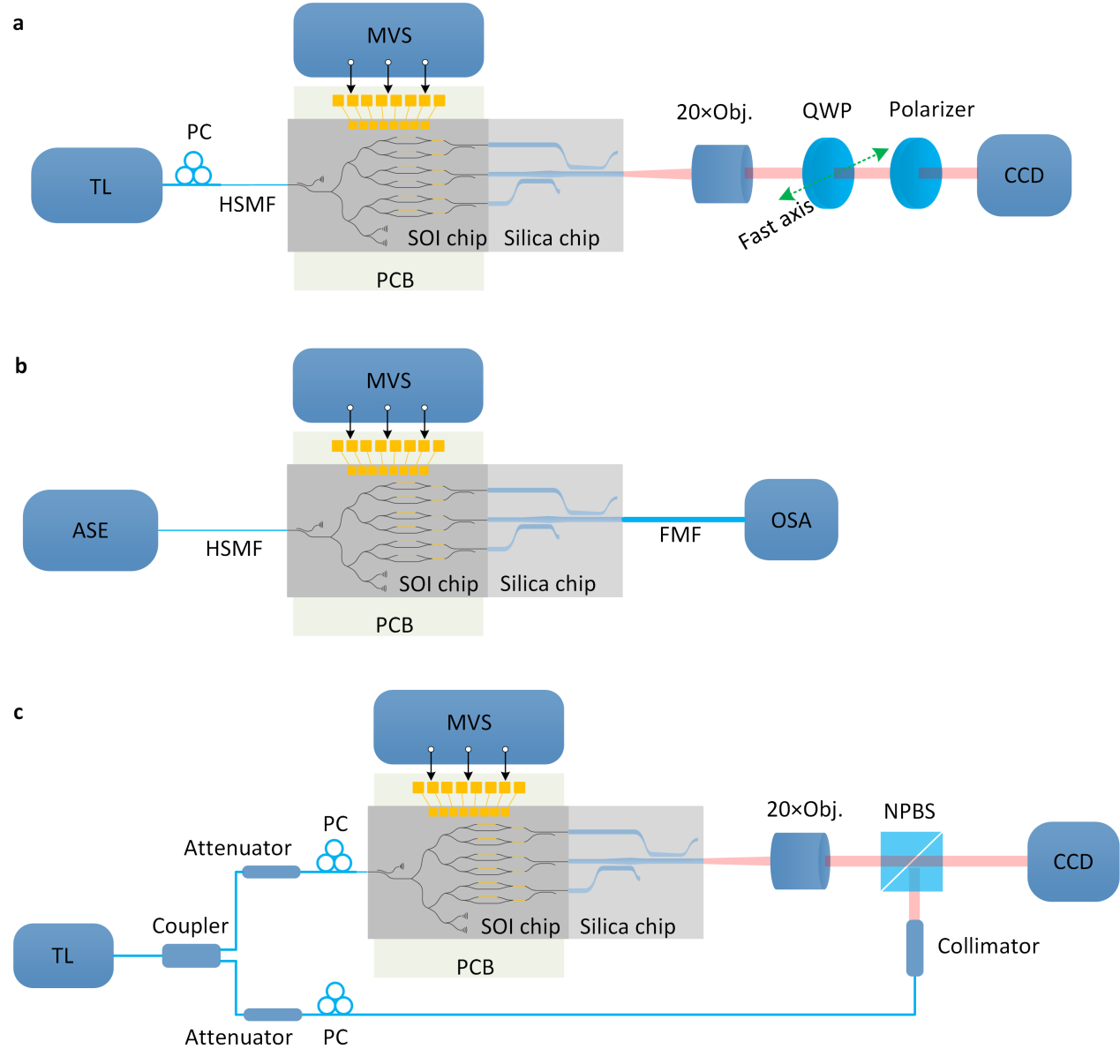


**Figure S3.** **Measurement experiment setup.** **(a)** Experiment setup for mode field detection, **(b)** Experiment setup for the measurement of mode transmissions. **(c)** The experiment setup for detecting the OAM beams. TL: tunable laser, ASE: amplified spontaneous emission, MVS: multichannel voltage source, PCB: printed circuit board, HSMF: high-NA single mode fibre, PC: polarization controller, MVS: multi-channel voltage source, FMF: few-mode fibre, OSA: optical spectrum analyzer, QWP: quarter-wave plate, NPBS: nonpolarizing beam splitter, CCD: charge-coupled device.
